# Reevaluating the Importance of Modified Ultrafiltration in Contemporary Pediatric Cardiac Surgery

**DOI:** 10.3390/jcm7120498

**Published:** 2018-12-01

**Authors:** Vladimir Milovanovic, Dejan Bisenic, Branko Mimic, Bilal Ali, Massimiliano Cantinotti, Ivan Soldatovic, Irena Vulicevic, Bruno Murzi, Slobodan Ilic

**Affiliations:** 1 Department of Cardiac Surgery, University Childrens Hospital, 11 000 Belgrade, Serbia; dejanbisenic@gmail.com (D.B.); irenavu@hotmail.com (I.V.); 2 East Midlands Congenital Heart Centre, University Hospitals of Leicester, Leicester LE39QB, UK; brankomimic@gmail.com (B.M.); alisbilal@gmail.com (B.A.); 3 Institute of Clinical Physiology, Fondazione G. Monasterio CNR-Regione Toscana, 56100 Pisa, Italy; massimiliano.cantinotti@ftgm.it; 4 School of Medicine, University of Belgrade, 11 000 Belgrade, Serbia; soldatovic.ivan@gmail.com (I.S.); bogoyu@gmail.com (S.I.); 5 Fondazione G. Monasterio CNR-Regione Toscana, 54100 Massa, Italy; murzi@ftgm.it

**Keywords:** modified ultrafiltration, conventional ultrafiltration, pediatric cardiac surgery, clinical outcomes

## Abstract

Objective(s): Modified ultrafiltration has gained wide acceptance as a powerful tool against cardiopulmonary bypass morbidity in pediatric cardiac surgery. The aim of our study was to assess the importance of modified ultrafiltration within conditions of contemporary cardiopulmonary bypass characteristics. Methods: Ninety–eight patients (overall cohort) weighing less than 12 kg undergoing surgical repair with cardiopulmonary bypass were prospectively enrolled in a randomized protocol to receive modified and conventional ultrafiltration (MUF group) or just conventional ultrafiltration (non-MUF group). A special attention was paid to forty-nine neonates and infants weighing less than 5 kg (lower weight (LW) cohort). Results: Post-filtration hematocrit was significantly higher in the MUF group for both cohorts (overall cohort *p* = 0.001; LW cohort *p* = 0.04), but not at other time points. During the postoperative course, patients in the MUF group received fewer packed red blood cells, (overall cohort *p* = 0.01; LW cohort *p* = 0.07), but required more fresh frozen plasma (overall cohort *p* = 0.04; LW cohort *p* = 0.05). There was no difference between groups in hemodynamic state, chest tube output, duration of mechanical ventilation, respiratory parameters, duration of intensive care unit, and hospitalization stay. Conclusions: If conventional ultrafiltration provides adequate hemoconcentration modified ultrafiltration does not provide additional positive benefits except for reduction in blood cell transfusion, This, however, comes at the cost of needing more fresh frozen plasma. Of particular importance is that this also applies to infants with weight bellow 5 kg where modified ultrafiltration was supposed to have the greatest positive impact.

## 1. Introduction

Cardiopulmonary bypasses (CPB), particularly in pediatric cardiac surgery, significantly contributes to the development of postoperative morbidity. Pediatric patients due to CPB develop a systemic inflammatory response syndrome (SIRS) which increases total body water and may result in multi-organ dysfunction. Most significant characteristics of CPB that trigger SIRS are hypothermia, hemodilution, anticoagulation, nonpulsatile blood flow, and exposure of blood to nonendotheilazed surfaces [1,2].

Ultrafiltration (UF), during and after CPB, is an important tool which mitigates these side effects. Standard pediatric UF techniques are conventional ultrafiltration (CUF) and modified ultrafiltration (MUF). CUF implies UF during CPB, whereas MUF is performed after CPB discontinuation. These techniques are not mutually exclusive but rather complementary. First described by Naik et al. [1] in 1991, MUF has become the standard practice in vast majority of cardiac centers [2]. In the last 20 years numerous clinical studies have demonstrated that MUF can be effective in improving clinical outcomes. Reported benefits include: improved hemodynamic [3,4,5,6] and respiratory function [7,8,9,10], decreased chest tube output [11,12,13,14], reduced need for blood product transfusion [13,15,16], as well as increased hematocrit (Hct) [1,6,13,15,16,17], plasma proteins [15,16,17], and platelets (Plt) [15,16].

During the last decade, greater knowledge about CPB related pathology has led to great advances in technology. Patients are exposed to less hemodilution and foreign surface resulting in reduced inflammation and post CPB edema [18,19]. There is also a reduced dependence on hypothermia [20]. With these advancements, controversy regarding the optimal UF policy has become more relevant. Apart from potentially greater volume removed MUF does not have any proved benefit over CUF [21]. New retrospective studies demonstrate that elimination of MUF from simplified miniaturized CPB circuit have not led to negative outcomes [18]. Nevertheless there are no prospective studies that could validate clinical importance of MUF with the improved CPB characteristics. With our experience of significant reduction of hypothermia usage and amelioration of CPB circuit, we speculated that CUF alone would be sufficient as UF strategy. We hypothesized that utilization of MUF is not associated with better clinical outcomes. Thus, the aim of this prospective, randomized study is to reevaluate the importance of MUF in these new conditions with a special attention to neonates and infants weighing less than 5 kg.

## 2. Experimental Section

### 2.1. Study Design

The study includes 98 prospectively randomized patients weighing less than 12 kg who underwent CPB for surgical repair of congenital heart disease at cardiothoracic department of University Children Hospital between 1 April 2016 and 1 September 2017. The cut-off of 12 kg was chosen to ensure homogeneity since at our institution all patients with weight less than 12 kg receive a blood in CPB prime. The Exclusion criteria included: active noncardiac disease that was expected to compromise postoperative recovery, previous sternotomy (which may influence blood loss), patients requiring pre CPB blood transfusion, preoperative mechanical ventilator support and ongoing corticosteroid therapy. Two patients were excluded from the study due to non-compliance. Study protocol was approved by the University Children Hospital Ethical committee (26/203) and informed consent was obtained from the children’s parents. This study has not been registered with a public trial registry because it represents the summaries of standard clinical treatments. With a random numbers table, patients were allocated to receive CUF and MUF (MUF group) or CUF alone (non-MUF group). The trial was not blinded for theatre staff, however it was masked for the majority of outcome assessors (intensive care unit staff, laboratory technicians).

### 2.2. Anesthesia, Surgery, and CPB

Standardized institutional anesthetic and pediatric perfusion protocols were used in all patients, the absence of MUF in Non MUF group was the only change in perfusion practice.

Invasive arterial and central venous pressures, electrocardiogram, rectal and/or nasopharyngeal temperature, inspiratory and expiratory gas concentrations, and pulse oximetry were continuously monitored. The anesthetic induction agent chosen was sodium thiopental, and sevofluran, the muscle relaxation agent used was vecuronium. Fentanyl and sevofluran were used for analgesia and maintenance of anesthesia respectively. Preoperative steroids were not given. Anticoagulation was established with initial heparin dose of 400 IU/kg and additional heparin was given during CPB to maintain activated clotting time greater than 480 s.

CPB circuit components, setup and prime were standardized. Two types of oxygenators were used: Sorin Kids D-100 (Sorin Group, Mirandola (MO), Italia) for neonates and Medtronic Affinity Pixie (Ann Arbor, MI, USA) for infants. The CPB prime contained buffered Ringer-lactate solution, 20% human albumin (50 mL), 20% manitol (maximum 2.5 mL/kg), methylprednisolone (30 mg/kg), and heparin 4000 IU/kg. Erythrocytes (one unit) were added to achieve a Hct of about 35% during CPB initiation.

Nonpulsatile CPB was established with standard aortic-bicaval canulation with a flow rate maintained at 125–200 mL/kg/min, the mean arterial pressure was kept above 35 mm Hg. On CPB, the Hct was maintained at or above 30%. Continuous mixed venous oxygen saturation, as well as in line arterial blood gas monitoring (CDI 500, Terumo Cardiovascular) were standard on all cases to ensure adequacy of perfusion.

Myocardial preservation was achieved with cold blood cardioplegia (1:4 proportion of hyperkalaemic solution and blood). Most of the procedures were performed under normothermia or mild hypothermia down to 32 °C, four cases, however, required isolated cerebral and myocardial perfusion or deep hypothermic circulatory arrest. Milrinone routinely, and adrenalin if needed, were infused for CPB discontinuation.

Decisions regarding postoperative inotropic support were based on degree of hemodynamic stability and clinical judgment. After achieving hemodynamic stability, mechanical ventilatory support and sedation were weaned. Patients were extubated when they were able to sustain adequate spontaneous respiration and require minimal oxygen support as reflected by normal arterial blood gas levels.

Blood gas analysis of serial blood samples were obtained with a blood gas analyzer (GEM primer 3000, Instrumentation laboratory, Bedford, MA, USA) at following time points: after anesthesia induction (T1), 5 min after given type of UF cessation (T2), immediately after ICU admission (T3), and 6 h after ICU admission (T4). Except for the patients extubated within 6 h of arrival to the ICU fraction of inspired oxygen (FiO2) and mean airway pressure (MAP) were collected at the same time points. Parameters of gas exchange capacity (oxygen index—OI and respiratory index—RI) were calculated according to the succeeding formulas:

OI = MAP × FiO2/PaO2
(1)

where PaO2 is the partial oxygen blood pressure.

RI = P (A−a) O2/PaO2
(2)

Postoperative labs were drawn within first 15 min upon arrival in the ICU.

Vasoactive inotrope score (IS) was calculated using the equation of Gaies et al. [22] at five time points: post filtration (IS 1), arrival to the ICU (IS 2), 4 h post-op (IS 3), 12 h post-op (IS 4), and 24 h post-op (IS 5).

### 2.3. Ultrafiltration

UF (both CUF and MUF) was established with the DHF0.2 (LivaNova, London, UK) hemoconcentrator throughout study period.

Pre-bypass ultrafiltration (PBUF) was done as a standard procedure. CUF was performed throughout CPB with suction to the effluent side (−120 mm Hg), with aims of removing excess volume from the CPB circuit and to maintaining Hct at or above 30%. Crystalloids or packed red blood cells PRBC were added as necessary to provide adequate volume (dilutional ultrafiltration).

After separation from CPB, arteriovenous MUF was initiated. MUF was performed via cardiopegia system, with every custompack having a Y line for hemofilter outlet port with plastic clamps on each line. This setting allowed for an easy switch from CUF to MUF.

Blood was removed from the patient through aortic cannula, than pumped through hemofilter, warmed by heat exchanger in cardioplegia circuit and returned back to the patient through the cardioplegia delivery circuit which was attached to the venous cannula in the right atrium.

The flow rate through the hemofiltration circuit was set initially at 10–15 mL/kg/min and then gradually increased, if tolerated by the patient, to a maximum of 20 mL/kg/min, adjusted to maintain appropriate central venous pressure and mean arterial pressure.

Once adequate flow through the MUF circuit has been established, the filtrate side of the circuit was opened. Suction (−120mm Hg), was also applied to the effluent side of hemoconcentrator during MUF. MUF was performed until all residual volume from venous reservoir and oxygenator had been re-infused.

### 2.4. Statistical Analysis

Results are presented as counts (percent) or means (standard deviations), depending on data type. *T* test and Mann-Whitney *U* test were used to assess significant differences between groups regarding numerical data, while Pearson chi-squared test was used for nominal data. A linear mixed model was used to assess significant differences between groups regarding numerical variables with repeated measurements. All *p* values less than 0.05 were considered significant. All data were analyzed using SPSS 20.0 (IBM corp.) and R for Windows 3.3.1

## 3. Results

### 3.1. Demographics

Ninety-eight children were enrolled in the study (overall cohort), and forty-nine of them weighed less than 5 kg (lower weight (LW) cohort)). Demographic characteristics for both cohorts are presented in Table 1. There were no statistically significant differences between the two groups in age, weight, body surface area, minimum core temperature, CPB time, aortic cross-clamping time or Aristotle basic score. Most of patients in both cohorts were operated in normothermia and mild hypothermia. Only four neonatal patients, equally distributed between both groups, underwent surgical repair with deep hypothermia—below 26 °C (three for interrupted aortic arch repair and one for Norwood procedure for hypoplastic left heart syndrome). There was one death in the MUF group in a child with Swiss cheese type of multiple VSD and stenotic parachute mitral valve. There were no known adverse events related to MUF in any patient.

### 3.2. CPB and Ultrafiltration

Table 2 shows CPB and ultrafiltration data for both cohorts. In both cohorts ultrafiltrate volume was significantly greater in the MUF group than in the Non MUF group (*p* = 0.05 for both cohorts). Likewise CPB prime volume was, also, significantly greater in the MUF group than in the Non MUF group (*p* = 0.04 and *p* = 0.01 respectively)). Urine output, fluid added during CPB, including PRBC’s, did not differ between groups.

### 3.3. Hematocrit (Hct)

**Figure 1:**
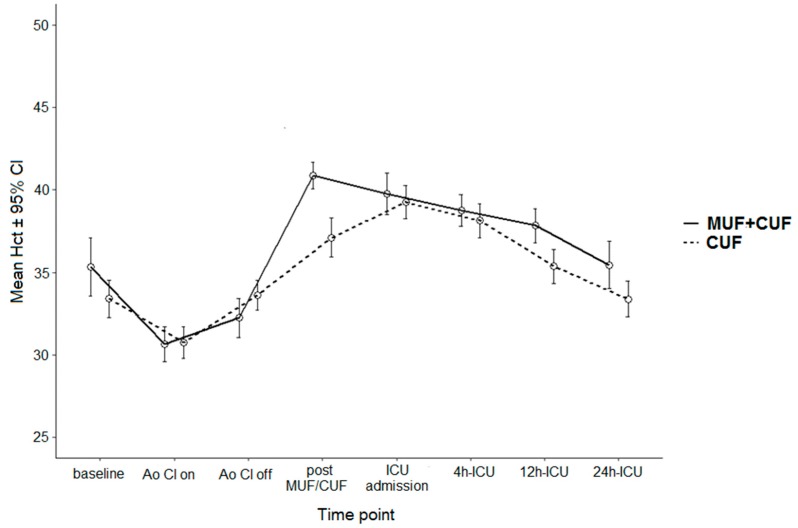
Hematocrit data for overall cohort—Ao Cl on—cross clamp on; Ao Cl off—cross clamp off; MUF—modified ultrafiltration; CUF—conventional ultrafiltration, ICU—intensive care unit.

**Figure 2:**
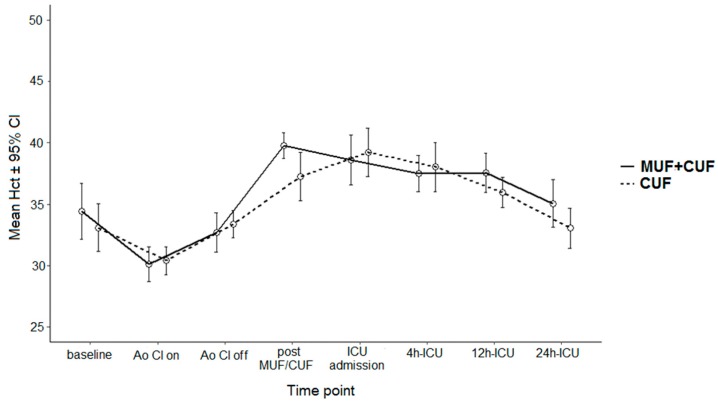
Hematocrit data for lower weight cohort—Ao Cl on—cross clamp on; Ao Cl off—cross clamp off; MUF—modified ultrafiltration; CUF—conventional ultrafiltration, ICU—intensive care unit.

Hct data are presented in Table 3. In both cohorts post filtration Hct was significantly higher in the MUF group versus the Non MUF group (*p* = 0.001 and *p* = 0.04 respectively) (In the overall cohort Hct was significantly higher in the MUF group within 24 h of ICU admission (*p* = 0.04). There were no differences in Hct values between the groups at 5 min after clamping of the aorta, 5 min after aortic declamping, upon ICU admission, as well as after 4 and 12 h in the ICU. Of note, the Hct change from baseline to 24 h in ICU did not differ between the groups in both cohorts. Figure 1 and Figure 2 show the Hct values at each time point for overall and LW cohort.

### 3.4. Postoperative Blood Requirement and Transfusion Data

Postoperative transfusion frequency, transfusion data as well as post-operative labs and chest tube output are presented in Table 4 and Table 5. The proportion of patients who received PRBCs was significantly smaller in the MUF group in the overall cohort (*p* = 0.01), and in the LW cohort the difference nearly reached statistical significance (*p* = 0.07). On the other hand, the proportion of patient who received FFP was significantly smaller in the non-MUF group for overall cohorts (*p* = 0.04) and LW cohort (*p* = 0.05), respectively. There was no difference between groups in both cohorts regarding the proportion of patients who received PLT and Cryo. In relationship to the volume of blood products in those patients who receive them in the overall cohort the MUF group received significantly more PRBC than the non-MUF group (*p* = 0.01), while in the LW cohort the volume of transfused PLT was also significantly greater in the MUF group (*p* = 0.01). Post op labs as well as chest tube output (the first 24 h in the ICU) did not differ between the groups in both cohorts.

### 3.5. Respiratory Parameters and Duration of Mechanical Ventilation

Table 6 shows various respiratory parameters. There was no significant difference in P (A−a) O2, OI and RI between the groups in the overall cohort. In the LW cohort P (A−a) O2 at the third time point was significantly smaller in the non-MUF group (*p* = 0.05), overall delta P (A−a) O2, however, did not differ between the groups. Additionally, in the LW cohort, RI at the third time point was significantly smaller in the MUF group (*p* = 0.02), once again without difference in delta RI. The duration of postoperative mechanical ventilatory support did not differ between the groups in both cohorts

### 3.6. Vasoactive Inotrope Scores, Intensive Care Unit (ICU), and Hospital Stay

Vasoactive inotrope score data, duration of intensive care unit and hospital stay are presented in Table 7. There was no difference in the cohorts over length of stays in both the ICU and hospital setting. In the overall cohort inotropic score (IS) at different time points was lower in the non-MUF group, but this did not reach statistical significance. Furthermore, in the LW cohort at all but one time period, IS, was lower in the MUF group, but this lacked statistical significance. Finally the delta IS values did not differ between the groups in the overall cohort and LW cohort.

## 4. Discussion

The present study aimed to reevaluate the importance of MUF as a part of combined ultrafiltration strategy on early clinical outcomes in infants undergoing pediatric cardiac surgery within conditions of decreased use of hypothermia and mitigation of hemodilution. As far as we are aware, this is first prospective study that evaluates the significance of MUF within these new CPB characteristics. Previous studies that compared CUF to CUF+MUF were characterized by significant utilization of hypothermia and deep hypothermic circulatory arrest [6,11,18,21]. Deep hypothermia with circulatory arrest, isolated myocardial and cerebral perfusion within our group of patients are reserved exclusively for children necessitating reconstruction of the ascending aorta and aortic arch (e.g., Hypoplastic heart syndrome, interrupted aortic arch). Furthermore, reduction of CPB circuit together with possibility of continuous in line Hct and arterial blood gas monitoring (CDI 500, Terumo Cardiovascular, Ann Arbor, MI, USA) resulted in reduction of the hemodilution degree. In our prospective, randomized study we paid special attention to neonates and infants weighing less than 5 kg where the benefits of MUF are expected to be more pronounced.

When Naik [1] first applied MUF, the basis of his approach was the removal of a greater volume of fluid than they had been able to achieve with CUF. Over time the improvement in CPB and CUF techniques have resulted with significant increase in CUF efficiency [18,21]. Many early pro-MUF studies involved significant hemodilution and compared MUF groups to control groups that received no ultrafiltration at all [1,3]. In the present day CPB management without any ultrafiltration is unthinkable and would not reflect actual clinical practice [2,18]. Another major problem with interpretation of study findings is the variety of techniques that have been used for ultrafiltration, as well as retrospective nonrandomized nature of many of these reports [21].

When evaluating our CPB and ultrafiltration data it is important to understand the reason for difference in priming volume for both cohorts. Priming volume in the MUF group was significantly greater due to the necessity to flush out the cardioplegia solution from the line before starting MUF. This results in shifting as much as 100 mL of blood from the venous reservoir to the cardioplegia line, which must be replaced with other volume. Trying to add as little volume as possible during CPB, our perfusionists have a policy of having greater priming volume that will be pretreated with pre-bypass ultrafiltration; we prefer this over adding additional blood or crystalloids before and during MUF.

In our study, volume of ultrafiltration obtained was, as expected, significantly greater in MUF groups for both cohorts. Previous studies indicate that the benefit of ultrafiltration correlate with the volume of filtrate removed [3,4,13]. When compared to other relevant studies our extent of ultrafiltration is higher than in the reports of Kotani et al. [6] and Thomson et al. [17], but not as aggressive as in studies of McRobb et al. [18] or Williams et al. [23]. Nevertheless, there are only two studies [17,18] that provide data regarding priming volume, volume of fluid and packed red blood cells added during CPB, and urine output during CPB. It is difficult to compare the aggressiveness of both CUF and MUF without this information.

When evaluating Hct and transfusion data, it is important to consider that there were no differences between the groups in volume of packed red blood cells added in priming and during CPB. Apart from a few other reports [6,18] and our study, target Hct during and after CPB was significantly lower in previous studies [10,11,14,15,20]. The higher post filtration Hct in the MUF group reflects MUF’s ability to further increase hemoconcentration. Hence, it is not surprising that the proportion of patients receiving packed red blood cells in the post-op period was greater in the non-MUF group for both cohorts (statistically significant for overall cohort only). Absence of difference in post-op delta hematocrit values comes at the expense of higher transfusion risk for non-MUF patients. Despite this, patients in the MUF overall group still have significantly higher Hct after 24 h in the ICU. It is important to highlight the consequence of lower transfusion rate in the MUF group. Transfusion leads to substantial changes in the immune system of the child and increases the occurrence of infections and recurrence of malignancies [24]. Furthermore, transfusion is occasionally complicated by transfusion-related acute lung injury [25,26].

Greater proportion of patients receiving fresh frozen plasma in the MUF group for both cohorts is difficult to interpret, especially in the light of lower transfusion rate of PRBC in MUF patients. Furthermore there were no differences in chest tube output and postoperative labs between the groups. The main indications for FFP transfusion during study period were bleeding and abnormal coagulation labs. We can speculate that MUF patients had greater initial drainage that was rather treated with FFP in the conditions of relatively higher postoperative Hct level. Obviously, this speculation is not valid enough to draw any strong conclusion. Still, McRobb et al. [18] also reported higher transfusion rates of cryoprecipitate in neonatal MUF group, also without the difference in chest tube output and fibrinogen levels between the groups. On the other hand, previous reports have suggested that MUF increased the concentration of coagulation factors and that it attenuated the coagulopathy associated with CPB [16]. However, hemostasis difficulty after CPB does not have a simple pathologic cause; on the contrary, multiple factors are involved [23].

Consistent with results of most previous studies [6,7,10,15,18,21] utilization of MUF did not contribute to the improvement of respiratory function. If the improvement in post CPB pulmonary function is predominantly caused by the ability to remove excess fluid, it seems that both ultrafiltration techniques are equally efficient. As Mahmoud et al. [10] have pointed out, the advantages of MUF on pulmonary function might be of limited duration only, rather than sustained for a long postoperative period. Nevertheless, the difference in a few respiratory parameters in the LW cohort might indicate higher sensitivity of neonatal/infant lung on the type of ultrafiltration applied. In his study Kotani et al. [6] speculated that using MUF in neonates is not robust enough to shorten the duration of ventilation, but might enable avoidance of maximal ventilatory support and prevention of subsequent possible ventilator-induced lung injury in the immediate postoperative period.

Similar to other recent studies [17,18,21] we found no difference in hemodynamic status between the groups in both cohorts. However, over a long period of time MUF has been attributed with positive impact to post CPB hemodynamic [3,4,5,6,11,13,15]. This was mainly explained with MUF ability to reduce post CPB myocardial edema and restore normal myocardial function [3,4]. Nevertheless, some authors speculate that improvement in hemodynamic during MUF is caused by the rapid increase in a blood viscosity after weaning from CPB with relatively low Hct [18]. Although in our study post filtration Hct was significantly higher in MUF group it did not result in lower IS. Furthermore, at most time points in the overall cohort IS was lower in Non MUF patients. While post filtration Hct in Non MUF group was significantly lower than in MUF group, the absolute value is still high (mean value over 37%). This finding supports the thesis that higher post CPB Hct provides better hemodynamic state. As previously and consistently reported [7,10,14,15,18,21], MUF failed to contribute to shortening of ICU and hospital length of stay.

## 5. Conclusions

In conclusion, our result demonstrate that if conventional ultrafiltration is sufficiently aggressive to provide adequate hemoconcentration, modified ultrafiltration does not generally provide positive effects in terms of clinical outcomes when compared to CUF. Of note, there was a reduced need for red blood cell transfusion in our MUF group, this, however, was opposed by a greater need for fresh frozen plasma. Of particular importance, these observations also apply to infants with weight below 5 kg where we had previously thought that modified ultrafiltration was supposed to have the greatest benefits, Further studies incorporating recent advances in the CPB technology are required to reinforce these results, especially in the population of neonates and small infants with various congenital heart lesions.

### Study Limitations

It is important to note some of the study limitations. As the selection of ultrafiltration strategy is undoubtedly linked to the characteristics of CPB, our results cannot be necessarily applicable to other centers with different CPB conducts. Patient populations may also differ. Potential reason for a lack of difference in outcomes could be attributed to relatively small sample size in the low weight cohort. One of our aims is to share our experience and stimulate further studies that will reevaluate ultrafiltration strategies within these new CPB characteristics. Moreover, as the level of important inflammatory mediators were not measured, we do not know if there is a difference in MUF and CUF’s abilities in removing cytokines within the new CPB conditions.

## Figures and Tables

**Table 1:** Demographics and operative data.

	MUF	Non MUF	*p*-Value
*N*	49	49	
Age (days)	160 ± 159	196 ± 169	0.23
Weigh (kg)	6.2 ± 2.9	6.7 ± 3.0	0.38
BSA (m2)	0.3 ± 0.2	0.3 ± 0.1	0.60
Minimum core temperature (°C)	32.9 ± 4.3	34.2 ± 2.1	0.15
CPB time (min)	101.4 ± 46.8	91.6 ± 47.3	0.24
X-CL time (min)	51.4 ± 27.3	52.6 ± 26.1	0.83
Aristotle basic score	7.6 ± 2.1	7.4 ± 2.1	0.64
LW cohort			
*N*	26	23	
Age (days)	44 ± 31	45 ± 32	0.81
Weigh (kg)	3.9 ± 0.8	4.1 ± 0.7	0.55
BSA (m2)	0.2 ± 0	0.2 ± 0	0.72
Minimum core temperature (°C)	32.0 ± 5.5	33.8 ± 2.3	0.34
CPB time (min)	111.9 ± 47.1	109.9 ± 60.0	0.63
X-CL time (min)	54.8 ± 28.1	61.6 ± 31.4	0.96
Aristotle basic score	8.4 ± 2.4	8.3 ± 2.1	0.82

MUF—modified ultrafiltration; N—number; LW—lower weight; BSA—body surface area; CPB—cardiopulmonary bypass; X-CL—cross clamp.

**Table 2:** CPB and ultrafiltration data.

	MUF	Non MUF	*p*-Value
*N*	49	49	
CPB Prime volume (mL/kg)	76.7 ± 31.2	64.9 ± 24.4	0.04
Ultrafiltrate volume (mL/kg)	127.7 ± 66.5	98.3 ± 42.7	0.05
Fluid added during CPB (mL/kg)	97.4 ± 50.2	89.50 ± 52.3	0.44
PRBC’s added during CPB (mL/kg)	26.8 ± 12.2	33.7 ± 22.3	0.45
Urine output during CPB (mL/kg)	8.5 ± 13.5	7.0 ± 14.5	0.14
LW cohort			
*N*	26	23	
CPB Prime volume (mL/kg)	97.7 ± 26.1	81.6 ± 21.7	0.01
Ultrafiltrate volume (mL/kg)	170.9 ± 57.6	133.8 ± 33.4	0.05
Fluid added during CPB (mL/kg)	111.5 ± 36.5	120.4 ± 52.0	0.49
PRBC’s added during CPB (mL/kg)	33.5 ± 13.9	42.4 ± 43.8	0.97
Urine output during CPB (mL/kg)	10.0 ± 14.7	9.4 ± 18.9	0.42

CPB—cardiopulmonary bypass; PRBC’s—packed red blood cells.

**Table 3:** Hematocrit data.

	MUF	Non MUF	*p*-Value
*N*	49	49	
Baseline (%)	35.3 ± 6.5	33.4 ± 4.1	0.08
Cross clamp on (%)	31.6 ± 3.4	31.7 ± 2.6	0.8
Cross clamp off (%)	32.2 ± 4.1	33.6 ± 3.0	0.06
Post CUF/MUF (%)	40.8 ± 3.8	37.1 ± 4.9	0.001
ICU admission (%)	39.7 ± 5.2	39.2 ± 4.1	0.58
4 h ICU (%)	38.7 ± 4.8	38.1 ± 3.9	0.5
12 h ICU (%)	37.8 ± 4.4	35.3 ± 4.4	0.08
24 h ICU (%)	35.4 ± 5.1	33.3 ± 3.9	0.04
Delta HCT 1–8	0.12 ± 7.3	−0.64 ± 5.0	0.58
LW cohort			
*N*	26	23	
Baseline %	34.4 ± 6.5	33.0 ± 4.8	0.41
Cross clamp on (%)	31.1 ± 3.9	31.3 ± 2.1	0.76
Cross clamp off (%)	32.6 ± 4.3	33.3 ± 1.9	0.47
Post CUF/MUF (%)	39.7 ± 3.7	37.2 ± 4.9	0.04
ICU admission (%)	38.6 ± 5.7	39.2 ± 4.8	0.69
4 h ICU (%)	37.5 ± 4.6	38.0 ± 4.3	0.67
12 h ICU (%)	37.5 ± 3.7	35.9 ± 3.0	0.11
24 h ICU (%)	35.0 ± 4.0	33.0 ± 3.6	0.09
Delta HCT 1-8	−0.16 ± 6.67	−0.62 ± 5.06	0.79

CUF—conventional ultrafiltration; MUF—modified ultrafiltration; ICU—intensive care unit; HCT—hematocrit.

**Table 4:** Postoperative transfusion frequency.

	MUF	Non MUF	*p*-Value
*N*	49	49	
PRBC	22 (44.9%)	36 (73.5%)	0.01
FFP	17 (34.7%)	8 (16.3%)	0.04
Cryo	11 (22.5%)	7 (14.3%)	0.36
PLT	8 (16.3%)	5 (10.3%)	0.46
LW cohort			
*N*	26	23	
PRBC	18 (69.3%)	21 (91.3%)	0.07
FFP	12 (46.1%)	5 (21.7%)	0.05
Cryo	9 (34.7%)	7 (30.4%)	1.00
PLT	6 (23.1%)	5 (21.7%)	0.52

PRBC—packed red blood cells, FFP—fresh frozen plasma, Cryo—cryoprecipitate, PLT—platelets.

**Table 5:** Transfusion data, post-op labs, and chest tube output.

	MUF	Non MUF	*p*-Value
*N*	49	49	
Peri-op PRBC (mL/kg)	28.33 ± 20.15	17.77 ± 12.79	0.01
Peri-op FFP (mL/kg)	14.8 ± 15.6	9.1 ± 3.2	0.43
Peri-op cryo (mL/kg)	9.8 ± 5.2	8.5 ± 6.3	0.30
Peri-op platelets (mL/kg)	11.7 ± 6.3	4.6 ± 1.2	0.09
ICU platelet count (× 103/uL)	115.3 ± 33.1	127.6 ± 44.5	0.12
INR	1.7 ± 0.4	1.8 ± 0.3	0.69
Fibrinogen (g/L)	2.0 ± 0.8	1.7 ± 0.6	0.14
PT (sec)	21.0 ± 4.9	21.8 ± 3.5	0.36
APTT (sec)	39.1 ± 14.4	41.40 ± 10.0	0.37
Chest tube output (mL/kg)	26.6 ± 21.6	25.2 ± 21.4	0.60
LW cohort			
*N*	26	23	
Peri-op PRBC	31.3 ± 21.0	23.3 ± 13.8	0.16
Peri-op FFP (mL/kg)	17.1 ± 17.8	9.4 ± 3.4	0.55
Peri-op cryo (mL/kg)	10.6 ± 4.8	9.0 ± 6.7	0.25
Peri-op platelets (mL/kg)	14.8 ± 4.0	4.7 ± 1.4	0.01
ICU platelet count (× 103/uL)	116.8 ± 32.9	130.4 ± 54.4	0.29
INR	1.9 ± 0.5	1.9 ± 0.3	0.80
Fibrinogen (g/L)	1.8 ± 0.8	1.6 ± 0.6	0.46
PT (sec)	22.6 ± 5.7	22.8 ± 3.5	0.89
APTT (sec)	44.9 ± 13.3	45.9 ± 8.9	0.75
Chest tube output (mL/kg)	33.8 ± 22.7	30.5 ± 23.6	0.47

PRBC—packed red blood cells; FFP—fresh frozen plasma; Cryo—cryoprecipitate; INR—international normalized ratio; PT—prothrombin time; APTT—*activated* partial thromboplastin *time*.

**Table 6:** Effects on gas exchange capacity and ventilation time.

	MUF	Non MUF	*p*-Value
P (A−a) O2 1	0.55 ± 0.38	0.59 ± 0.29	0.52
P (A−a) O2 2	0.44 ± 0.21	0.44 ± 0.21	0.92
P (A−a) O2 3	0.53 ± 0.24	0.50 ± 0.23	0.53
P (A−a) O24	0.57 ± 0.23	0.58 ± 0.22	0.70
Delta P (A−a) O2 1–4	0.39 ± 0.13	0.30 ± 0.20	0.65
OI 1	4.5 ± 5.4	3.3 ± 3.4	0.21
OI 2	4.5 ± 4.4	4.1 ± 3.2	0.53
OI 3	3.4 ± 3.4	3.5 ± 3.6	0.83
OI 4	3.9 ± 4.1	2.9 ± 1.5	0.59
Delta OI 1–4	2.7 ± 3.1	2.1 ± 2.6	0.39
RI 1	2.3 ± 3.2	1.5 ± 2.3	0.32
RI 2	2.2 ± 2.7	2.0 ± 1.7	0.90
RI 3	1.8 ± 2.5	1.8 ± 2.1	0.48
RI 4	1.4 ± 2.2	1.4 ± 2.7	0.72
Delta RI 1–4	0.13 ± 4.39	−0.54 ± 2.93	0.28
Ventilation time (h)	46.5 ± 99.3	33.7 ± 55.1	0.53
LW cohort			
P (A−a) O2 1	0.46 ± 0.31	0.50 ± 0.24	0.68
P (A−a) O2 2	0.40 ± 0.21	0.32 ± 0.16	0.14
P (A−a) O2 3	0.47 ± 0.22	0.34 ± 0.18	0.05
P (A−a) O24	0.50 ± 0.22	0.46 ± 0 .20	0.55
Delta P (A−a) O2 (1–4)	0.05 ± 0.35	0.18 ± 0.31	0.15
OI 1	5.9 ± 6.8	4.5 ± 4.5	0.57
OI 2	4.7 ± 3.3	5.1 ± 3.7	0.67
OI 3	3.5 ± 2.5	5.3 ± 4.4	0.11
OI 4	3.3 ± 1.7	3.2 ± 1.5	0.96
Delta OI (1–4)	3.4 ± 1.2	3.8 ± 2.1	0.41
RI 1	2.8 ± 3.4	2.1 ± 2.9	0.61
RI 2	2.4 ± 2.7	2.9 ± 1.8	0.13
RI 3	2.0 ± 2.6	2.9 ± 2.5	0.02
RI 4	1.8 ± 2.2	1.9 ± 2.4	0.50
Delta RI (1–4)	4.57 ± −0.75	3.97 ± −2.30	0.41
Ventilation time (h)	82.1 ± 123.8	75.0 ± 130.4	0.84

P (A−a) O2—alveolar-arterial oxygen pressure difference; OI—oxygen index; RI—respiratory index.

**Table 7:** Vasoactive inotrope scores, ICU, and hospital length of stay.

	MUF	Non MUF	*p*-Value
*N*	49	49	
IS 1 (post filtration)	7.1 ± 5.7	6.4 ± 9.2	0.10
IS 2 (ICU admission)	7.1 ± 5.7	6.7 ± 10.7	0.20
IS 3 (4 h)	7.3 ± 6.0	6.7 ± 10.7	0.11
IS 4 (12 h)	6.6 ± 5.4	6.5 ± 10.7	0.29
IS 5 (24 h)	5.9 ± 5.3	6.4 ± 10.8	0.64
Delta IS (1–5)	1.01 ± 2.72	0.03 ± 11.3	0.56
ICU LOS (days)	6.8 ± 10.2	5.0 ± 7.0	0.58
Hospital LOS (days)	15.4 ± 14.0	14.3 ± 14.2.0	0.43
LW cohort			
*N*	26	23	
IS 1	8.2 ± 6.2	6.7 ± 3.4	0.19
IS 2	8.1 ± 6.3	9.3 ± 14.8	0.41
IS 3	8.6 ± 6.8	9.3 ± 14.8	0.16
IS 4	7.5 ± 6.3	9.3 ± 14.8	0.69
IS 5	7.0 ± 6.4	9.9 ± 14.8	0.37
Delta IS (1–5)	1.0 ± 1.5	−2.5 ± 12.8	0.17
ICU LOS (days)	11.2 ± 12.5	8.9 ± 9.1	0.83
Hospital LOS (days)	23.8 ± 14.8	24.2 ± 16.1	0.59

IS—inotropic score; ICU—intensive care unit; LOS—length of stay.
